# A school-based intervention to promote physical activity among adolescent girls: Rationale, design, and baseline data from the *Girls in Sport *group randomised controlled trial

**DOI:** 10.1186/1471-2458-11-658

**Published:** 2011-08-19

**Authors:** Anthony D Okely, Wayne G Cotton, David R Lubans, Philip J Morgan, Lauren Puglisi, Judy Miller, Jan Wright, Marijka J Batterham, Louisa R Peralta, Janine Perry

**Affiliations:** 1 Interdisciplinary Educational Research Institute, University of Wollongong, Northfields Avenue, Wollongong, 2522, Australia; 2 Faculty of Education, University of Wollongong, Northfields Avenue, Wollongong, 2522, Australia; 3 Faculty of Education and Social Work, University of Sydney, Parramatta Road, Sydney, 2006, Australia; 4 Educational Research Institute, University of Newcastle, University Drive, Callaghan, 2308, Australia; 5 School of Education, University of Newcastle, University Drive, Callaghan, 2308, Australia; 6 School of Education, The University of New England, Meredith Road, Armidale, 2351, Australia; 7 Centre for Statistical and Survey Methodology, University of Wollongong, Northfields Avenue, Wollongong, 2522, Australia; 8 School of Education, Southern Cross University, Hogbin Drive, Coffs Harbour, 2450, Australia

## Abstract

**Background:**

Physical activity levels decline markedly among girls during adolescence. School-based interventions that are multi-component in nature, simultaneously targeting curricular, school environment and policy, and community links, are a promising approach for promoting physical activity. This report describes the rationale, design and baseline data from the *Girls in Sport *group randomised trial, which aims to prevent the decline in moderate-to-vigorous intensity physical activity (MVPA) among adolescent girls.

**Methods/Design:**

A community-based participatory research approach and action learning framework are used with measurements at baseline and 18-month follow-up. Within each intervention school, a committee develops an action plan aimed at meeting the primary objective (preventing the decline in accelerometer-derived MVPA). Academic partners and the State Department of Education and Training act as critical friends. Control schools continue with their usual school programming. 24 schools were matched then randomized into intervention (n = 12) and control (n = 12) groups. A total of 1518 girls (771 intervention and 747 control) completed baseline assessments (86% response rate). Useable accelerometer data (≥10 hrs/day on at least 3 days) were obtained from 79% of this sample (n = 1199). Randomisation resulted in no differences between intervention and control groups on any of the outcomes. The mean age (SE) of the sample was 13.6 (± 0.02) years and they spent less than 5% of their waking hours in MVPA (4.85 ± 0.06).

**Discussion:**

*Girls in Sport *will test the effectiveness of schools working towards the same goal, but developing individual, targeted interventions that bring about changes in curriculum, school environment and policy, and community links. By using community-based participatory research and an action learning framework in a secondary school setting, it aims to add to the body of literature on effective school-based interventions through promoting and sustaining increased physical activity participation among adolescent girls.

**Trial Registration Number:**

Australia and New Zealand Clinical Trials Register (ANZCTR): ACTRN12610001077055

## Background

Physical inactivity is one of the leading modifiable risk factors for mortality and morbidity among adults, responsible for an estimated 3.2 million deaths in 2004 [1]. Patterns of activity in adulthood are often established during adolescence [2], making this an important period for promoting physical activity. Moreover, prevalence rates show that adolescent girls are less active than boys [3,4] and that activity declines more precipitously among girls during adolescence [5,6].

To address this, several school-based interventions have been developed to promote physical activity among adolescent girls. These have targeted modifying the formal curriculum (such as physical education classes [7] and school sport [8,9], school environment [10] and community links [11]. Collectively, these three components comprise a Health Promoting Schools Framework [12], which aims to intervene across multiple levels of influence in a students' life in a consistent and integrated way.

A limitation with these interventions is that few have implemented strategies across all three Health Promoting Schools components [13] and several used a 'one size fits all' approach where all schools in a treatment group implement the same intervention. Given the heterogeneity of schools in terms of their goals, size, geographical location, teacher expertise, socioeconomic status, and culture, this may result in some elements of an intervention being inappropriate for the school. An approach gaining popularity is one in which all schools follow the same process and work towards the same goal, but use different strategies to achieve these goals based on the different needs and resources in their school [14]. This process involves using formative data about the school's social and physical environment to develop an action plan that prioritises where and how change needs to be made. Using an action learning model, schools form small teams or committees who take responsibility for setting the priorities and implementing them through a school action plan. Researchers act as critical friends who support the school's committee. This approach has been used successfully in a project to reduce unsafe sexual behaviours [15]. The efficacy of this approach for promoting physical activity has not been examined.

The *Girls in Sport *Action Research Project is part of an overarching state-wide initiative called the Premier's Sporting Challenge [16]. This Challenge seeks to promote sport and physical activity participation of children and youth attending government primary and secondary schools in the state of New South Wales, Australia. The GIS project specifically targets girls in Grade 8 in 2009 and Grade 9 in 2010 with the goal of creating school and community environments that encourage and support the full involvement of girls in physical activity, including sport, physical education, recreation and leisure time activities [17]. This will be accomplished through a multi-component school- and community-based intervention, informed by research and evaluated to develop a framework which can be adapted for use in other schools. This paper describes the rationale, design, and baseline characteristics of the *Girls in Sport *group randomised controlled trial.

## Methods/Design

The study was funded by the New South Wales (NSW) Department of Education and Training (who are responsible for all government schools) through a competitive tender process and is registered with the Australian New Zealand Clinical Trials Registry (ACTRN12610001077055). The University of Wollongong Human Research Ethics Committee approved the study protocol (HE08/296).

### Setting and population

This study is a partnership between the School Sport Unit of the NSW Department of Education and Training (who is responsible for the promotion and implementation of school sport within the Department) and NSW universities including the University of Wollongong, University of Sydney, University of Newcastle, University of New England, and Southern Cross University. As the structure of school sport in NSW is unique, a brief explanation is provided. In NSW, government secondary schools are required to provide students in Years 7-10 with at least two hours of planned physical activity each week [18]. This activity can be achieved through a combination of physical education and compulsory school sport. In regards to school sport, each school can develop and conduct their own unique sport program according to their student needs and interests, school resources and teacher expertise, availability of transport and community facilities. These school sport programs may include inter and intra-school competitive sport, recreational sport, fitness and/or skill development activities. Generally schools implement their sport program pattern in a number of ways: integrated, traditional, or staggered. Where an integrated sport pattern occurs, at least 80 additional minutes are to be incorporated into the Personal Development, Health and Physical Education (PDHPE) program. In traditional and staggered sport patterns, 80-120 minutes are allocated every week specifically to sport. Traditional patterns occur on one afternoon each week, whereas in a staggered pattern certain year groups will have sport allocated at certain times. In traditional and integrated sport patterns most of a school's teaching staff, including non-physical education trained teachers, are allocated sport as part of their teaching load [8].

### Aims of the study

The primary aim of the study was to test if an 18-month school-based intervention targeting school sport and physical education (through the formal curriculum), school ethos (including policies and school breaks such as lunchtime), and links with the local community, could prevent the decline in objectively measured physical activity compared with matched control schools.

### School and participant selection

The NSW Department of Education and Training emailed all principals of secondary schools in NSW (N = 500), calling for Expressions of Interest to participate in the project. Interested schools were asked to contact the Department and then complete a profile for their school which was used to pair-match schools prior to randomization. This profile requested information about the school's population (number of boys and girls), proportion of students from non-English speaking and Aboriginal and Torres Strait Islander backgrounds, number of years teaching experience of the physical and health education staff, and how school sport and physical education were organised in the school. Schools were then matched based on these criteria in addition to their type of school (for example, girls only, central school [found in rural areas where primary and secondary schools are combined on the one site under the same school executive], and technology high schools [which help prepare students for the changing needs of today's society through providing specialist options in robotics, computing, and rural and marine technologies]) and their geographic location. A member of the research team was then assigned to be a 'critical friend' at each school. This involved working with the school as it collected and interpreted their own data and assisting with the development, implementation, and evaluation of the school's action plan [19]. Within each school, all girls in Grade 8 (second Grade of secondary schooling) were invited to participate in the study.

### Randomization and study design

This was a group randomized controlled trial with school as the unit of randomization. Each matched pair of schools was randomly allocated to the intervention or control group using a computer-based random number producing algorithm. This was undertaken by a researcher independent of the project and then communicated to the research team who informed each school of its allocation. Due to the timeframe of the study, formative research was required to be conducted in the intervention schools in Term 4 (October to December 2008). This meant randomization needed to occur prior to baseline data collection.

### Inclusion and exclusion criteria

To be eligible for the study, schools were required to not be involved in any other physical activity-related research projects and to confirm that they consented to being randomized to either the intervention or control group if they were selected for the project. If selected in the intervention group, schools needed to commit to developing an action plan, forming a school committee, attending workshops, organizing students for data collection days and putting the project in the school management plan. Girls needed to be formally enrolled in Grade 8 within the participating schools and provided written consent from themselves and their parent(s)/guardian(s) prior to participation. If a student or their parent/guardian did not consent, they were still able to participate in the intervention activities but did not participate in data collection.

### Formative research

The formative research aimed to identify from the target group (Grade 8 girls) their needs and interests and the schools and community facilitators and barriers to their participation in physical activity. Interviews were held with relevant staff (including PE and non-PE staff and a member of the school executive), informal interviews with groups of boys, and focus groups with girls in Grade 8, during October and November 2008. A member of the research team attended along with the project manager, who collected the data. In addition, girls and PE staff were asked to map community physical activity facilities and opportunities on provided maps of the school and local community. Observations of PE lessons, recess and lunchtime activities were also conducted. Results suggested that there were two main reasons schools were interested in participating in the study. These were the chance to revitalise sport in their school and the opportunity to engage particular groups of girls who were currently not participating in PE or school sport. From the perspective of staff, the reasons for girls not participating in school sport were grouped into three themes: 1) the current structure of school sport (which lacked variety and limited choice, with those who were less skilled and confident being the last to choose a sport); 2) the lack of resources; and 3) the lack of expertise among non-PE staff who supervised school sport. Among the girls, the main reasons for their non-participation were similar to those reported by staff, with additional barriers identified including the 'dominating' behaviours of boys during PE lessons and sport and the girls' perceived lack of skills and confidence in traditional PE and school sport.

Girls were asked what they would like in a school sport program to enhance their engagement and participation. They suggested the following: the opportunity to choose some of the activities (especially non-traditional ones such as yoga, self-defence, and dance); participate with their friends; motivated and knowledgeable staff; more modern sports uniforms; greater cooperative behaviour from boys; and higher levels of activity during sessions (less time spent sitting around). It was not surprising that these factors have been cited as motivators for physical activity among adolescent girls [8].

These formative data were then provided to each school as a report and school committees were asked to ensure they addressed as many of them as possible when developing their intervention strategies and action plans. For example, to give girls greater choice in the types of activities in which they could participate, staff were encouraged to survey girls to determine what activities they would like and then examine ways some of them could be integrated into their school sport programs. They were also advised of the importance of students being a part of the school committee so they had a 'voice' on the program in their school.

### Intervention

Individual schools, in conjunction with their critical friend, developed unique 18-month action plans to implement from mid 2009 to the end of 2010. The specific intervention strategies derived for each school was independently designed to achieve the overarching aim of the project. This aim was the same for each school, and was to prevent a decline in participation in moderate-to-vigorous intensity physical activity levels among girls over the course of the intervention. There were also six secondary aims that schools needed to work towards (see Table 1). Each school followed an identical process in developing their intervention. This involved: 1) forming an action learning team (Committee) within their school community; and 2) developing school-specific action plans in three areas based on the results of the formative research and on individual needs of the school. These areas were the formal curriculum, the school ethos and environment, and home/school/community links [12]. Schools were also asked to identify barriers to them meeting the outcomes identified in their action plans. Once these plans were written, each school commenced implementation, and were encouraged by researchers to continually reflect on their progress and modify the strategies where required. Support was also given to the schools in a variety of ways, including funding from the Department of Education and Training, an initial two-day training program, regular contact with the Girls in Sport project manager and research team, informal school surveys, as well as a two-day research symposium in February 2010.

**Table 1:** Outcomes for the Girls in Sport Intervention and Research Project

	Outcomes
*Primary Outcome*	
1.	Prevent a decline in MVPA levels of targeted girls
*Secondary Outcomes*	
2.	Greater awareness among participating schools and community sport and physical activity providers of the needs, interests and issues for girls
3.	Sport and physical activity programs that are better designed to meet these needs
4.	Functional links to community sport and physical activity facilities and services
5.	Improved confidence and self efficacy of participating girls
6.	Integrated opportunities for girls and boys to contribute to and influence decisions about sport and physical activity participation of girls
7.	More involvement in school and community sporting activities.

The 12 control schools will continue with their usual program and schooling without change. At the conclusion of the project these schools will receive training and materials related to the findings of the project.

### Measures

Data were collected within individual school settings. Baseline data were collected by trained measurement staff from February 2009 to June 2009. After this period, the intervention schools participated in the *Girls in Sport *program, while the control schools continued with their regular school sport programs. Subsequent follow-up data collection took place from July to December 2010. Data collectors were blinded to group allocation. Teachers and students at the paired intervention and control schools were also blinded towards their matched comparison school. To enhance the quality of the data across all collection sites, the research assistants were formally trained in standardized measurement procedures and protocols. Each research assistant was given a detailed manual, checklist and scripts to read when informing the participants about the measures. Data collectors checked for incorrectly completed questionnaires (i.e., pages or items not filled in) and invited participants to correct their mistakes or complete missing items. The teachers in the schools were also asked to follow up any absent participants.

#### Primary Outcome Measure

Objectively measured moderate-to-vigorous intensity physical activity was the primary outcome for the study. All participants wore an Actigraph accelerometer (7164 and GT1M models; Fort Walton Beach, FL) for seven consecutive days. This was attached to an adjustable elastic belt and worn over the right hip. Data were collected in 30-second epochs. The average number of minutes that the accelerometer was worn and the number of activity counts per minute (CPM) were calculated. Mean CPM as a summary measure of total physical activity in children has been validated against doubly labelled water [20]. Thirty-second activity counts were uploaded to determine the amount of time spent in light (LPA; 1.5-2.9 METs) moderate (MPA; 4-6.9 METs) and vigorous (VPA; ≥7 METs) physical activity during the monitoring period. Age-specific count ranges relating to the above intensity levels were based on prediction equations for energy expenditure [21]. Values were calculated for percentage of monitored time spent in light, moderate, and vigorous physical activity to account for variation in time spent wearing monitors. Participant data were included in analyses if accelerometers were worn for ≥600 minutes on ≥3 days [3].

All participants were asked to keep activity monitoring logs for the seven-day period when the accelerometers were being worn. Participants also received two text messages during the seven-day period. These text messages reminded the participants to keep wearing the accelerometers and to return them at the end of the seven-day period.

#### Potential mediators and moderators of physical activity behaviour change

The questionnaires used to measure potential mediators and moderators were administered in a secluded area on two separate days to increase the accuracy of responses and reduce participant burden. The first visit was when the accelerometers were distributed and the second when the accelerometers were collected approximately seven days later. On the first visit the enjoyment of physical activity and school sport, self-efficacy in physical activity, social support for physical activity, social support during school sport, strategies to increase physical activity, and school physical activity environment scales were administered. On the second visit, the physical self-concept and perceived importance of physical activity scales were administered.

Potential mediators assessed included enjoyment of physical activity and school sport, physical activity self-efficacy, social support for physical activity, social support during school sport, strategies to increase physical activity, school physical activity environment, physical self-concept and perceived importance of physical activity. We conducted our own validity and reliability of all scales. To do this, four schools were selected at random to complete the scales, approximately one week apart: Seventy-five students from two schools completed those administered on the first visit, and another 75 from the other two schools completed those administered on the second visit. Intra-class correlations were performed to assess test-retest reliability and Cronbach alphas were used to assess internal consistency. AMOS 17.0 (Small Waters Corp., Chicago IL) was used to assess the construct validity of the different measures using the baseline data. Model fit was assessed using multiple indices, including chi-square index, comparative fit index (CFI) and root mean square of approximation (RMSEA).

##### Enjoyment of Physical Activity and School Sport

General enjoyment of physical activity and specific enjoyment of school sport were measured using a modified version of the Physical Activity Enjoyment Scale (PACES) [22]. The S-PACES measure comprises seven negatively worded items from the original PACES instrument [23]. The items are rated on a 5-point Likert-scale with semantic anchors ranging from "disagree a lot" to "agree a lot". The stems used to cue responses in this study were "When I am active..." and "When I participate in school sport...". Sample items were: "It frustrates me", and "It's no fun at all". The test-retest reliability (ICC = 0.86, 95% CI = 0.77 to 0.91) and internal consistency (α = 0.90) of the PACES were good. Similarly, the internal consistency (α = 0.91) and test-retest reliability (ICC = 0.83, 95% CI = 0.73, 0.89) of the scale were also good. The model fit indices for enjoyment of physical activity in the study population were χ^2 ^= 142, *p *< 0.001, CFI = 0.97 and RMSEA = 0.09. The model fit indices for enjoyment of school sport were χ^2 ^= 246, *p *< 0.001, CFI = 0.96 and RMSEA = 0.12.

##### Physical Activity Self-Efficacy

Physical activity self-efficacy was measured using an eight-item questionnaire originally developed by Motl et al. [24]. Example items on the self-efficacy measure included "I can be physically active during my free time on most days no matter how busy my day is," and "I can be physically active during my free time on most days even if it is hot or cold outside". All eight items were rated on a five-point scale ranging from 1 (strongly disagree) to 5 (strongly agree). The reliability of the eight-item instrument was acceptable (ICC = 0.8, 95% CI = 0.85 to 0.94 and internal consistency ∝ = 0.78) and the model fit indices for the sample were χ^2 ^= 60, *p *< 0.001, CFI = 0.98 and RMSEA = 0.04

##### Social Support for Physical Activity

Social support was assessed using the Peer Support Scale developed by Prochaska, Rodgers and Sallis [25]. The instrument employed a 5-point Likert scale anchored by 1 (Never) and 5 (Daily). The participants were asked to report how many times during a typical week they received or gave various forms of support from/to their friends e.g. "Do you encourage your friends to do physical activities or play sport" or "Do your friends encourage you to do physical activities or play sport". The Peer Support Scale was found to have high internal consistency (α = .73) and good test-retest reliability (ICC = .86). The model fit indices for enjoyment of physical activity were good χ^2 ^= 13, *p *= 0.002, CFI = 0.99 and RMSEA = 0.07

##### Social Support during School Sport

Social support received during school sport was measured using a modified version of an existing scale [26]. The scale included five items relating to students' beliefs about the instruction and social support students received from teachers and instructors during school sport. Students responded to a 5-point Likert scale (1 = Strongly Disagree to 5 = Strongly Agree). 'During school sport my teacher/instructor...' was the common stem and included the following items: i) appears enthusiastic about school sport, ii) teaches me valuable movement skills, iii) participates in physical activity or sport with me, iv) makes the activity enjoyable, v) encourages me to participate in the activity and vi) demonstrates sound knowledge and understanding of the activity. The internal consistency (α = 0.82) and test-retest reliability (ICC = 91, 95% CI = 0.85, 0.94) values were acceptable. The model fit indices for scale in the study population were good χ^2 ^= 31, *p *< 0.001, CFI = 0.99 and RMSEA = 0.05

##### Behavioural Strategies to Increase Physical Activity

Behavioural Strategies used to increase physical activity were measured using an adapted version of a scale derived from self-management theory for use with college students [27]. Participants were asked how often they used various strategies to increase their motivation for physical activities e.g. "I do things to make physical activity more enjoyable", and "I set goals to do physical activities". The modified seven-item instrument used a 5-point Likert scale ranging from 1 (never) to 5 (very often) for responses. Test-retest reliability of the modified version were ICC = 0.93 (95% CI = 0.89 to 0.96) whilst internal consistency was α = 0.78. The model fit indices for physical activity behavioural strategies in the study population were χ^2 ^= 263, *p *< 0.001, CFI = 0.92 and RMSEA = 0.12.

##### School Physical Activity Environment

Participants were asked to rate the quality, accessibility and availability of the physical activity facilities at their school using a scale developed for the current study. An example of an item was "The physical activity facilities at my school are easily accessible to me". Options ranged from 1 (Strongly disagree) to 5 (Strongly agree). The test-retest reliability (ICC = 0.69, 95% CI = 0.51 to 0.81), internal consistency (α = 0.80) and model fit indices (χ^2 ^= 98, *p *< 0.001, CFI = 0.96 and RMSEA = 0.07) were adequate.

##### Children's Physical Self-Perception Profile including the Perceived Importance Profile

The participants' physical self-perceptions were examined using the Children's Physical Self-Perception Profile (C-PSPP) inventory [28]. The C-PSPP is a 30-item questionnaire which consists of five equally divided sub-domains: perceived sport competence, physical conditioning, body attractiveness, physical strength, and physical self-worth. Each item presents two alternative statements, from which the participants can select which one best represents themselves using "sort of true" or "really true". The factor validity of the PSPP has been supported across various age groups, including adolescents [29,30]. Test-retest reliabilities and internal consistencies for all sub-domains were > 0.80. The model fit indices for the different sub-domains of the C-PSPP were considered acceptable to good in the study population. The Perceived Importance Profile (PIP) is integrated into the PSPP and centres on the importance the participants attach to four of the PSPP sub-domains, physical self-worth is excluding. The PIP utilises the same scoring structure as the PSPP and incorporates two items to measure the importance of each of the four sub-domains. The internal consistency (α = 0.78) and test-retest reliability (ICC = 91, 95% CI = 0.86, 0.94) values for the PIP were acceptable. The model fit indices for the PIP scale in the study population were χ^2 ^= 216, *p *< 0.001, CFI = 0.90 and RMSEA = 0.09.

### Process evaluation

In a study such as *Girls in Sport*, where each school will implement an intervention that is slightly different in its context, it is important to thoroughly document what is implemented, and the social context in which this occurs, as level of implementation can directly influence the outcomes of the study. In *Girls in Sport*, each school was required to submit an action plan for 2009 and 2010. This action plan formed part of the overall school plan for the year. School plans provide a framework to drive change within a school over a 3-year period in areas such as student engagement and retention, teacher quality, and connected learning [31].

The action plan for this study took each of the overarching outcomes and asked schools to write down specific strategies they would undertake to achieve this target and how they would measure success in achieving it, along with who would be involved. The activities also needed to demonstrate the area of the Health Promoting Schools Framework they represented.

Each school's specific action plan was then reviewed by the research team and Department of Education and Training staff. Schools participated in monthly teleconferences with their research partner to share their progress towards the study outcomes, specifically, the implementation of their strategies and any barriers they were experiencing. Possible solutions were brainstormed and, if further assistance was required, the problems were revised at the teleconference held every three weeks between the research team and Department staff and possible solutions fed-back to the school.

At the end of each year, schools were asked to document their progress towards the study outcomes based on their implementation of their specific strategies. They also evaluated any barriers to implementation. Interviews were also conducted at the end of the intervention with each school committee, other staff, executive, girls and boys, to triangulate these data and assess the extent to which the strategies were implemented. Observations of school sport and lunchtime activities will also take place.

### Sample size justification

The primary analysis in this study will be conducted in SAS using a linear mixed model. The test of interest will be an F test with a 1 degree of freedom contrast therefore it is computationally convenient to use the t test to perform the sample size calculations. Murray [32] proposed a method of sample size estimation and published relevant intraclass correlations on which to base the estimate. In lieu of a reliable estimate of the intra class correlation for the primary outcome measure of activity CPM an estimate of 0.01 was used in the a priori calculations. Effect sizes and variance estimates, 77.51SD(102.92) cpm, which was 18.4% of the baseline mean, were obtained from a previous study [33]. Based on these figures, a model based on a critical t value of 2.228 (taking into consideration the matching of the schools) is obtained for estimates based on 12 schools per group. Variance estimates are adjusted for clustering as proposed in Murray [32]; in brief the standard error of the estimate in the usual t estimation is replaced by $\sqrt{\frac{2\left({\hat{\sigma }}_{m}^{2}+m{\hat{\sigma }}_{g}^{2}\right)}{mg}}$ where ${\hat{\sigma }}_{m}^{2}$ is the estimate of the unadjusted subject component of the variance, ${\hat{\sigma }}_{g}^{2}$ is the unadjusted school component of the variance, m is the number of subjects per school and g is the number of schools per intervention. Sample sizes as low as 10 participants per school completing the study provided adequate power (>80% power and P < 0.05). Given that the estimate of effect could be considered optimistic for the present design a more modest effect size (10% of baseline mean, 42.07 cpm) was also considered. It was also anticipated that group sizes would vary between schools and therefore the estimates were based on a harmonic mean of 30 participants per school completing [34]. With this conservative mean effect size and a harmonic mean sample size of 30 completing the study the power remains high (0.987).

### Statistical Analyses

#### Primary analyses

Statistical analysis of the primary outcome variable, percentage of time spent in MVPA, will be performed using a linear mixed model (PROC MIXED) is SAS. This model accounts for the hierarchical structure of the data. This is a standard statistical procedure for analysis of clustered datasets and the use of this methodology in school-based trials has been extensively documented by Murray [32]. Analysis of sedentary behaviour, light, moderate and vigorous activity will be performed in a similar manner. An advantage of the linear mixed model is that it incorporates all available data allowing for the analysis of partial datasets created when a participant drops out of the study or misses a study visit. Imputation of missing data will also be considered if missing data is substantial. This imputation will be performed using PROC MI and MIANALYSE. Sensitivity analyses will be performed. Mixed models will also be used to analyse all continuous secondary outcome variables.

#### Secondary analyses

Model Testing, Mediation and Moderation Analyses.

Two types of analyses will be conducted to explore the theoretical assumptions of the intervention. First, Social Cognitive Theory will be tested in AMOS using structural equation modelling. Hypothesized mediators of physical activity behaviour change will be examined using multilevel linear analysis and a product-of-coefficients test that is appropriate for cluster randomized controlled trials [35]. Potential moderators of the intervention effects (e.g. ethnicity, socio-economic status and type of school) will also be explored using multi-level modelling. The baseline analyses presented in the current paper are conducted using PROC MIXED (SAS V 9.0, SAS Institute, Cary NC) to adjust for the matched and clustered nature of the dataset [32].

## Results

**Figure 1:**
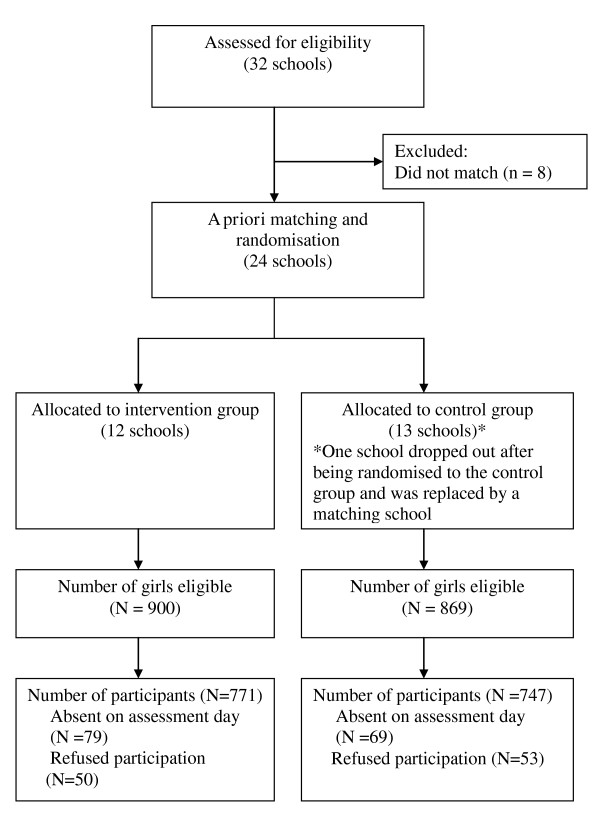
**Flow of schools and participants through the trial**.

A total of 32 schools from four geographical regions in New South Wales tendered an Expression of Interest for the study to the NSW Department of Education and Training and were assessed for eligibility. The four regions, outer Sydney metropolitan, Illawarra and South Coast, Hunter and New England, and the North Coast incorporated a range of socio-economic, urban and rural settings. From the original 32 schools, 8 were unable to be matched based on the *a priori *criteria, leaving 24 that were matched and randomly allocated to the intervention or control group. One of the schools withdrew after being allocated to the control group and was replaced with another school that was nominated by the NSW Department of Education and Training as a suitable school to be matched to the intervention school. The flow of schools and individual students through the enrolment and allocation stages of the study are displayed in Figure 1. Eighty-six percent of eligible girls completed baseline assessments, 771 in the intervention group and 747 in the control group. Table 2 shows the baseline characteristics of participants on demographic, outcome and mediator/moderator variables.

**Table 2:** Baseline demographic characteristics and outcome measures of *Girls in Sport *participants

	Intervention Group(1)	Control Group(0)	P	All
Age in years, mean (SE)	13.71 ± 0.04	13.48 ± 0.02	0.3512	13.60 ± 0.02
Physical activity* (n = 1199)				
Counts per minute, mean (SE)	428.40 ± 5.50	418.44 ± 5.22	0.2367	423.14 ± 3.79
Percentage time sedentary intensity	60.07 ± 0.32	61.22 ± 0.28	0.1210	60.83 ± 0.21
Percentage time light intensity	34.95 ± 0.27	33.95 ± 0.24	0.0655	34.26 ± 0.19
Percentage time moderate intensity	4.51 ± 0.08	4.32 ± 0.08	0.1482	4.38 ± 0.06
Percentage time vigorous intensity	0.455 ± 0.02	0.512 ± 0.02	0.6826	0.476 ± 0.01
Percentage time moderate-to-vigorous activity	4.97 ± 0.09	4.83 ± 0.09	0.3184	4.86 ± 0.06
Percentage of participants meeting recommended 60 mins of MVPA every day	1.9	1.1	0.253	1.5
Percentage of participants meeting recommended 60 mins of MVPA per day on average	10.6	9.6	0.682	10.1
Physical self-worth (n = 1467)	16.04 ± 0.14	16.40 ± 0.14	0.1798	16.22 ± 0.10
Sports competence (n = 1465)	15.42 ± 0.14	15.70 ± 0.13	0.2739	15.56 ± 0.10
Physical condition (n = 1468)	16.04 ± 0.14	16.38 ± 0.14	0.1389	16.21 ± 0.10
Body attractiveness (n = 1462)	14.02 ± 0.14	14.39 ± 0.15	0.2748	14.21 ± 0.10
Physical strength (n = 1461)	15.09 ± 0.13	15.35 ± 0.12	0.2002	15.22 ± 0.09
Physical activity confidence (n = 1510)	29.74 ± 0.16	29.88 ± 0.17	0.4675	29.81 ± 0.12
Enjoyment of physical activity (n = 1503)	29.77 ± 0.16	29.63 ± 0.16	0.5504	29.70 ± 0.12
Enjoyment of school sport (n = 1510)	28.06 ± 0.19	27.40 ± 0.20	0.0641	27.74 ± 0.14
Social support for physical activity (n = 1510)	12.54 ± 0.10	12.02 ± 0.11	0.0768	12.29 ± 0.08
Social support during school sport (n = 1503)	23.83 ± 0.16	23.65 ± 0.16	0.6594	23.75 ± 0.11
Strategies to increase physical activity (n = 1509)	25.32 ± 0.18	24.93 ± 0.19	0.3935	25.13 ± 0.13
School physical activity environment (n = 1508)	26.26 ± 0.16	25.88 ± 0.16	0.6952	26.07 ± 0.11
Perceived importance of physical activity (n = 1459)	22.42 ± 0.15	22.39 ± 0.15	0.8685	22.41 ± 0.11

Raw means are presented. Analysed intervention and control means and se are adjusted LS estimates from the PROC MIXED procedure in SAS V 9.2. The model used specifies randomisation group and pairing in the class statement as recommended by Murray [32]. Degrees of freedom are entered manually using the formula (randomisation groups-1)x(pairs-1) and in this study is equal to 11. Only those subjects who completed the specified questionnaire subdomains are included in this table with the numbers indicated in brackets (n). *accelerometer estimates include accelerometer type in the model.

We obtained useable accelerometry data from almost 80% of adolescent girls (79.1%). There were no statistically significant differences between the intervention and control groups on any of the variables. Although differences between the two groups approached statistical significance for three of the variables (percentage of time spent in light intensity physical activity, enjoyment of school sport, and social support for physical activity), from a clinical perspective they are not of any note. The sample demonstrated low levels of physical activity participation. Only 1.5% met current physical activity recommendations for adolescents of ≥60 minutes of MVPA every day [36]. Moreover, the girls spent 60% of their waking hours being sedentary and less than 5% in moderate-to-vigorous intensity physical activity (MVPA).

## Discussion

*Girls in Sport *is a group randomized trial which aims to test the effectiveness of an intervention developed by each school and that simultaneously targets school sport, environment and policy changes, and community links, to prevent a decline in MVPA among adolescent girls. Detailed formative research has guided the action plans developed by each school and these have been externally evaluated by the research team. Progression on these action plans is regularly maintained during the intervention through ongoing contact between school staff, the research team, and the NSW Department of Education and Training. As this is one of the first known school-based interventions to promote physical activity that has used a community-based participatory research approach and action learning framework, it is hoped the lessons learnt can inform others who are developing similar studies in the future.

To achieve acceptable response rates within each school (all >80% with many exceeding 90%) staff members of the school committee encouraged girls to return their consent forms and followed up any that were outstanding. Because these staff had a good rapport with the girls (many taught the girls or were their Year Advisor) they were able to explain in detail the study and what changes may result for these girls. Because the intervention involved all girls in the specific grade, there was no stigma associated with being involved.

Staff were also able to encourage girls to wear the accelerometer (many staff also wore them to enhance compliance) and stress the importance of keeping them on outside of school hours. Again, involving all girls reduced any stigma associated with others seeing the monitors. Schools were also encouraged to pay for release time for staff so they could organise the assessment times and locations for each class, chase up outstanding consent forms, and schedule interview times for girls and staff. This meant staff had time to devote to this and were not having to complete it on top of their other teaching and school commitments. Collecting the questionnaire data over two visits, one week apart, allowed the research team to collect the accelerometers on the second visit and also obtain any missing data from the first visit. Training school staff in how to show the girls the correct way to wear the monitor meant that they could distribute these to any girls who were absent during data collection. Conducting the questionnaires in a classroom with individual desks and away from other students (especially boys) facilitated quality data (less than 5% missing or completed incorrectly) as girls could feel assured that their responses would be treated confidentially.

There were no significant differences between groups at baseline for any of the variables. This shows that the matching of schools on demographic and other school-related factors prior to randomisation was successful and that collecting the baseline data after randomisation (which resulted in one school allocated to the control group withdrawing) did not result in any significant differences between groups. The matching of schools is an important and potentially effective way of maximising the chance of the groups being similar at the start of the intervention, minimising several potential threats to the internal validity of the study, especially differential threats [32].

Accelerometer data highlighted that girls spent 60% of their waking hours sedentary and only 5% in MVPA. These proportions are similar for both variables to those reported among a similar sample of 12-year-old girls as part of baseline measures for the TAAG study (55% sedentary and 3% MVPA, respectively) [37]. With regards to the raw accelerometry data (expressed as counts per minute), our value of 423.43 is similar to that reported for 12-15 year old adolescent girls in the NHANES study (381.60) [3].

This study will provide an opportunity to test a variety of mediators and moderators of physical activity behaviour. Few studies have assessed mediators of physical activity behaviour in youth interventions and this study will help provide insights into the mechanisms of behaviour change in a large sample of adolescent girls [38]. In addition, the majority of studies that explored potential mediators of intervention effects in youth have used self-report measures of physical activity. To the authors' knowledge only two studies have examined mediators in youth interventions using an objective measure of activity [39,40].

The development of positive physical self-perceptions may provide the foundation for future physical activity [41]. Previous studies have illustrated the importance of physical self-perceptions in explaining adolescents' physical activity behavior [42]. Adolescents' physical self-perceptions have been found to predict physical activity in longitudinal studies [43] and improve as a result of increased activity [44]. In the current study we will test the mediating and moderating effects of physical self-perception on physical activity.

## Conclusions

The *Girls in Sport *intervention is unique in that individual schools are primarily responsible for developing and implementing the intervention. By giving schools ownership of the intervention and the opportunity to develop strategies which meet the specific needs, interests and expertise within schools, it is hoped that these programs become embedded into the school culture, maximising their sustainability beyond the life of this evaluation project. It is also hoped that by each school forming a committee that includes at least one executive staff member, several staff from different faculty, and students, that the workload and responsibility is shared among the committee, again enhancing sustainability when staff (and students) move from the school.

## Competing interests

The authors declare that they have no competing interests.

## Authors' contributions

ADO, WGC, DRL, PJM, JM, JW obtained funding for the research. All authors contributed to developing the protocols and reviewing, editing, and approving the final version of the paper. ADO, WGC, DRL, PJM, LP, JM, JW, JP, LRP developed the intervention materials. ADO is the guarantor and accepts full responsibility for the conduct of the study and the integrity of the data. ADO is responsible for the accuracy of the preliminary data analysis.

## Pre-publication history

The pre-publication history for this paper can be accessed here:

http://www.biomedcentral.com/1471-2458/11/658/prepub
